# Nutritional Modulation of Gut Microbiota Alleviates Severe Gastrointestinal Symptoms in a Patient with Post-Acute COVID-19 Syndrome

**DOI:** 10.1128/mbio.03801-21

**Published:** 2022-03-07

**Authors:** Ying Wang, Guojun Wu, Liping Zhao, Weizheng Wang

**Affiliations:** a Department of Biochemistry and Microbiology, School of Environmental and Biological Sciences, Rutgers, The State University of New Jersey, New Brunswick, New Jersey, USA; b Center for Microbiome, Nutrition, and Health, New Jersey Institute for Food, Nutrition, and Health, Rutgers, The State University of New Jersey, New Brunswick, New Jersey, USA; c Division of Gastroenterology and Hepatology, Department of Medicine, Rutgers New Jersey Medical School, Newark, New Jersey, USA; University of Pittsburgh

**Keywords:** COVID-19, gastrointestinal symptoms, gut microbiota, long-hauler

## Abstract

With the increase in total coronavirus disease 2019 (COVID-19) infection cases, post-acute COVID-19 syndrome, defined as experiencing ongoing health problems 4 or more weeks after the first severe acute respiratory syndrome coronavirus 2 (SARS-CoV-2) infection, has become a new arising public health concern. As part of post-acute COVID-19 syndrome, gastrointestinal symptoms might be associated with dysbiosis of the gut microbiota, which has the potential to become a target for intervention. In this study, a patient with post-acute COVID-19 syndrome with long-lasting severe gastrointestinal symptoms was provided 2-month expanded access to a high-fiber formula with investigational new drug (IND) status developed to alleviate COVID-19-related symptoms by modulating the gut microbiota. Symptoms including severe “loss of appetite,” palpitation, and anxiety were significantly alleviated by the end of the intervention. The medication dosage for controlling nausea decreased during the intervention. The serum lipid profile, insulin level, and leptin level were improved compared to the baseline values. Significant structural changes of the patient’s gut microbiota and reduced microbial fermentation activity in the small intestine were found during the intervention. Eighteen amplicon sequence variants (ASVs) of the V4 region of the 16S rRNA gene significantly responded to this nutritional intervention. Six out of the 18 ASVs were also found to be negatively correlated with symptom severity/medication dosage. Five of the six ASVs (ASV0AKS_*Oscillibacter*, ASV009F_*Anaerofustis*, ASV02YT_*Blautia*, ASV07LA_*Blautia*, and ASV0AM6_Eubacterium hallii) were potential short-chain fatty acid (SCFA)-producing bacteria, which might be associated with the alleviation of symptoms. Our study indicates the feasibility of alleviating gastrointestinal symptoms in patients with post-acute COVID-19 syndrome by way of nutritional modulation of their gut microbiota.

## INTRODUCTION

While the coronavirus disease 2019 (COVID-19) pandemic caused by severe acute respiratory syndrome coronavirus 2 (SARS-CoV-2) has been slowly brought under control, the world is facing another problem, post-acute COVID-19 syndrome, which is characterized by persistent symptoms and/or delayed or long-term complications beyond 4 weeks from the onset of symptoms (1). It has been reported that 23.2% of COVID-19 patients experience post-acute COVID-19 syndrome with at least one symptom, and 10% might have health issues persisting for at least a year (2, 3). Gastrointestinal (GI) symptoms, including “loss of appetite,” nausea, vomiting, diarrhea, and abdominal pain, have been reported in COVID-19 patients (4). This might be caused by SARS-CoV-2 binding to the angiotensin-converting enzyme 2 (ACE2) receptors in intestinal epithelial cells, which might lead to infecting and damaging the epithelial cells, increasing gut inflammation, and affecting normal gut functions in digestion, etc. (5). As part of post-acute COVID-19 syndrome, GI symptoms such as nausea, abdominal pain, diarrhea have been reported to persist 6 months after acute COVID-19À (6), and 44% of hospitalized COVID-19 patients have been reported to experience GI symptoms 90 days after discharge (7). In some patients, severe GI symptoms might remain persistent for a much longer time.

Multiple studies have shown that the gut microbiota is closely related to COVID-19 prognosis (8–10). Patients with a dysbiotic gut microbiota have a higher risk of developing a more severe form of COVID-19 with catastrophic clinical outcomes due to (i) a higher level of baseline inflammation with a greater risk of developing immunopathology (11), (ii) more abundant opportunistic pathogens in the gut with a higher risk of developing complications, (iii) a lower abundance of beneficial bacteria that have previously been shown to have immunomodulatory functions, and (iv) more severe “leaky gut syndrome” if the viral infection also happens in the gut (12). Hospitalized COVID-19 patients had a gut microbiota significantly different from that of healthy controls, and the altered microbiota persisted 30 days after the patients cleared the infection (8, 10). A gut microbiota that remains dysbiotic after patients have recovered from COVID-19 might be a potential driving factor for ongoing symptoms after the depletion of SARS-CoV-2. For example, the overproduction of fermentation gas in the small intestine due to bacterial overgrowth might aggravate GI symptoms such as nausea and vomiting, etc. (13). Restoration of a healthy gut microbiota might help alleviate GI symptoms in patients with post-acute COVID-19 syndrome.

Our previous study showed that a high-fiber diet based on whole grains, traditional Chinese medicinal foods, and prebiotics (WTP diet) recovered a healthier gut microbiota by selectively increasing a group of short-chain fatty acid (SCFA) producers and decreasing proinflammatory and/or endotoxin-producing bacteria in patients with type 2 diabetes (14). SCFAs produced by gut bacteria can serve as an energy source for human colonocytes, mitigate inflammation, regulate satiety, and improve insulin production, etc. (14, 15). SCFA-producing bacteria were also shown to have an inverse correlation with post-acute COVID-19 syndrome (6). More importantly, akin to the tall trees of a closed forest, this group of SCFA-producing gut bacteria might work as the “foundation species” to structure and stabilize a healthy gut ecosystem. Members of these foundation species competitively thrive on a combination of dietary fibers as their energy source and produce SCFAs such as acetate and butyrate (14). They have been found to outcompete pathobionts in the patient’s gut, possibly by (i) acidifying the gut environment, (ii) producing antimicrobials, and (iii) taking up available ecological niches (14). A microbiota-targeted formula that contains a large amount of dietary fibers with diverse physicochemical structures was developed to recapitulate the modulating effect of the WTP diet on the gut microbiota to promote beneficial SCFA-producing bacteria and restore a healthier gut ecosystem. This formula has been given the investigational new drug (IND) status (IND 149631) and is currently being used in an ongoing phase 2 clinical trial for COVID-19 patients with acute infection (ClinicalTrials.gov identifier NCT04540406).

In this study, a female (patient LH01) between 55 and 60 years old with a history of Graves’ disease, well-controlled hypertension, hyperlipidemia, and prediabetes was infected by SARS-CoV-2 in April 2020. Her Graves’ disease was treated with radioactive iodine three times in 2003 with postprocedural hypothyroidism on a stable levothyroxine (Synthroid) supplement. She tested positive for COVID-19 in April 2020, with symptoms including shortness of breath, cough, nausea, and loss of appetite. She was considered a mild-to-moderate COVID-19 case and prescribed with in-home observation. Ten days later, she was diagnosed with left lower lobe pneumonia at urgent care, and amoxicillin, azithromycin (Z-pak), and cough medication were prescribed. However, the symptoms persisted after the treatments. Azithromycin and hydroxychloroquine treatments were then prescribed in May 2020. Her respiratory symptoms improved after treatment, while her GI symptoms became worse and persisted, which may be related to the gastrointestinal adverse effects of azithromycin and hydroxychloroquine (16, 17). Her case was complicated by elevated transaminase levels as well as increased abdominal pain and palpitations later. She was found to have gallbladder dyskinesia as evidenced by an abnormal hepatobiliary iminodiacetic acid (HIDA) scan showing delayed gallbladder emptying with a maximum gallbladder ejection fraction of 15% after cholecystokinin administration. Cholecystectomy was performed to alleviate her abdominal pain and nausea, which, however, failed to do so. She was put on dicyclomine for functional pain, pantoprazole for gastritis/gastroesophageal reflux disease (GERD), ondansetron (Zofran) for nausea/vomiting, and paroxetine for depression/anxiety. None of the treatments mentioned above were able to resolve her symptoms. She was a medical worker and was unable to return to her job because of the severe and persistent symptoms that could not be explained by an alternative diagnosis other than post-acute COVID-19 syndrome. Since she had COVID-19 infection, her quality of life was significantly hampered by severe persistent symptoms such as being unable to eat due to loss of appetite and nausea, anxiety, and constipation for over a year. Unfortunately, although she received many conventional treatments, including surgery, none of them were effective in her case.

Both patient LH01 and her gastroenterologist agreed with the hypothesis that the ecological changes in the gut microbiota induced by this high-fiber formula might help alleviate the patient’s post-acute COVID-19 syndrome. After obtaining approval from the institutional review board (IRB), consent from the patient, and IND 155864 by the FDA, patient LH01 was treated with the high-fiber formula for 2 months via the single-patient expanded-access program. To monitor her clinical and microbiota changes during the intervention, we collected data on her daily symptoms and medication intake together with time series breath, blood and fecal samples. Her GI symptoms were significantly alleviated with concomitant changes of the gut microbiota.

## RESULTS AND DISCUSSION

### Nutritional alleviation of post-acute COVID-19 syndrome.

During the 2-month intervention, the participant was instructed to slowly increase the dosage of the high-fiber formula at the beginning of the intervention to avoid potential discomfort due to the sudden increase in fiber intake. Basically, the participant consumed 1 dose/day in the first 2 days, 2 doses/day in the next 9 days, and 3 doses/day from day 12.

**FIG 1:**
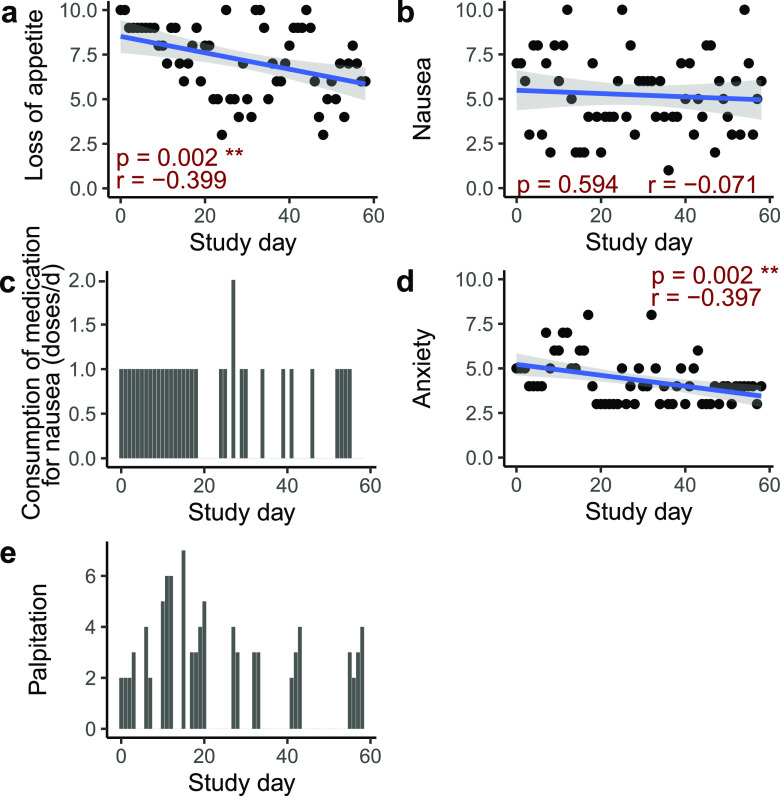
Severity of symptoms and medication intake decreased during the intervention. (a and b) Correlations between the severity of loss of appetite and nausea and days of intervention. (c) Bar plot showing that the frequency of medication intake for nausea decreased during the intervention. (d) Correlations between the severity of anxiety and days of intervention. (e) Bar plot showing that the frequency of palpitation occurrence decreased during the intervention. Symptoms were evaluated on a scale of 0 (none) to 10 (severe). The general linear regression line is shown as a blue line in panels a, b, and d. The 95% confidence interval is shown as a gray area around the regression line. The Pearson correlation was calculated, and the *P* value and *r* value are shown in dark red in each panel. Asterisks indicate significance (**, *P* < 0.01).

We monitored the participant daily to collect data on symptom severity, medication intake, and dosage of high-fiber formula consumption. General linear regression analysis showed that the severity of loss of appetite was significantly decreased (Fig. 1a). The severity of nausea showed a decreased trend (Fig. 1b). Since the medication intake for nausea (Fig. 1c) was prescribed to be taken as needed, the decreased frequency of medication intake for nausea indicated an improvement of nausea as well. The severity of anxiety and the frequency of palpitation occurrence decreased during the intervention (Fig. 1d and e).

**FIG 2:**
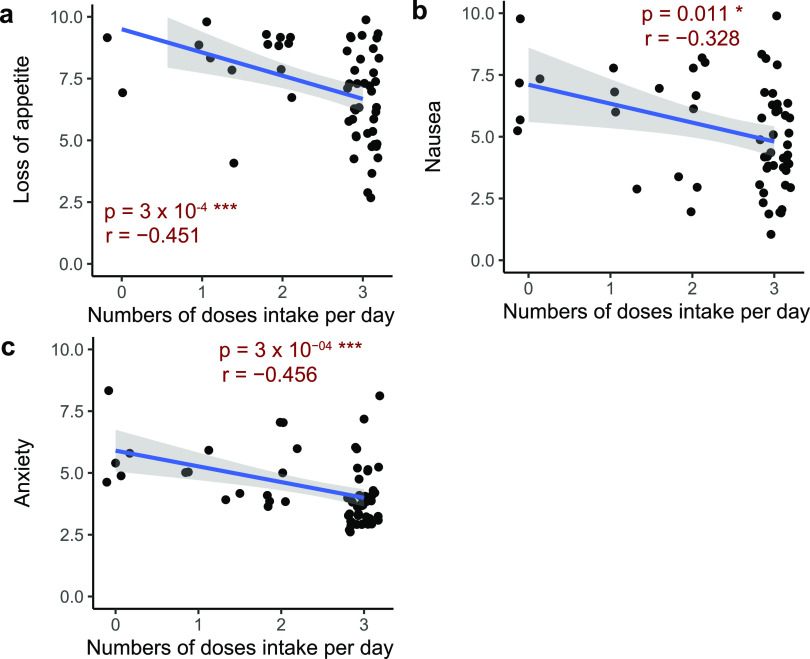
Severity of symptoms is alleviated with increased consumption of the fiber formula. Correlations between the severities of loss of appetite (a), nausea (b), and anxiety (c) and the consumption of the high-fiber formula were determined. Daily symptom severity and high-fiber formula consumption data collected from the 2-month intervention are plotted as points. Symptoms were evaluated on a scale of 0 (none) to 10 (severe). The general linear regression line is shown as a blue line. The 95% confidence interval is shown as a gray area around the regression line. The Pearson correlation was calculated, and the *P* value and *r* value are shown in each panel. Asterisks indicate significance (*, *P* < 0.05; ***, *P* < 0.001).

A dosage effect was also detected using general linear regression analysis. The severity of nausea, loss of appetite, and anxiety all significantly negatively correlated with the dosage, indicating that the high-fiber formula alleviated symptom severity more effectively with 3 doses/day than with 1 and 2 doses/day (Fig. 2a to c). All of the above-described results indicate that the consumption of the high-fiber formula alleviated the symptom severity in this post-acute COVID-19 syndrome case.

Blood sample-based clinical parameters, which are related to liver and kidney function and glucose and lipid metabolism, etc., were measured at days 9, 30, and 58 (Table 1). Previous blood work data (4 months prior to the intervention) were retrieved from the participant’s medical record as the baseline. Regarding liver function, the alkaline phosphatase (ALP) level was higher than the normal range before the intervention, and it became normal at the end of the intervention. Acute liver injury shown as elevated transaminase levels has been reported in approximately two-thirds of severe COVID-19 cases (18). In this case, elevated liver enzymes were still found 8 months after the first infection (i.e., 4 months before the intervention). Other liver function markers such as alanine transaminase (ALT) and aspartate transaminase (AST) were within normal ranges at all time points but also showed a decreased trend at the end of the intervention. All of the above-described results indicated an improvement of liver function after the intervention. The fasting insulin level decreased, while the fasting blood glucose and HbA1c levels stayed stable during the intervention. This is consistent with our previous study showing that the high-fiber diet could improve glycemic control and alleviate insulin resistance (14). Leptin, a hormone functioning in regulating food intake and energy balance, decreased during the intervention, indicating an improvement of energy homeostasis, which is consistent with our previous study showing that a high-fiber diet improved leptin resistance (19, 20). The overall lipid panel, including cholesterol, high-density lipoprotein (HDL) cholesterol, low-density lipoprotein (LDL) cholesterol, very-low-density lipoprotein (VLDL) cholesterol, and triglycerides, decreased during the intervention, indicating an improvement of the patient’s metabolic syndrome, which is consistent with the cholesterol-lowering effect of dietary fiber shown previously (21). This could be mediated by the role of the high-fiber formula in stimulating the excretion of bile acids, altering cholesterol absorption, and affecting cholesterol synthesis by SCFAs that are produced by the gut microbiota (22–24).

**TABLE 1:** Blood-based clinical parameters before, during, and after the 2-month intervention^*a*^

Parameter	Value				
	Baseline (4 mo prior)	Day 9	Day 30	Day 58	Normal range
ALP (IU/L)	173 ↑	205 ↑	197 ↑	144	44–147
ALT (U/L)	15	14	15	12	7–55
AST (U/L)	27	29	30	23	8–45
Total bilirubin (mg/dL)	0.2	0.2	<0.2	0.3	<1.2
Total protein serum (g/dL)	7.1	7.2	7	6.8	6.0–8.3
Albumin/globulin ratio	1.7	1.8	1.9	1.7	1.1–2.5
Albumin (g/dL)	4.5	4.6	4.6	4.3	3.5–5.5
Total globulin (g/dL)	2.6	2.6	2.4	2.5	2.0–3.9
BUN (mg/dL)	11	9	11	14	6–24
BUN/creatinine ratio	11	10	12	13	10–20
EGFR	74	79	78	66	>60
Serum glucose (mg/dL)	92	91	92	88	<140
Insulin (μIU/mL)	NA	13.8	13.4	11.1	2.6–24.9
Serum leptin (ng/mL)	NA	121.2 ↑	113.1 ↑	91.7 ↑	9.1–50.4
Cholesterol (mg/dL)	NA	184	165	145	<200
HDL cholesterol (mg/dL)	NA	76	72	57 ↓	≥60
LDL cholesterol (mg/dL)	NA	86	74	70	<100
VLDL cholesterol (mg/dL)	NA	22	19	18	2–30
Triglycerides (mg/dL)	117	128	110	98	<150

a ALP, alkaline phosphatase; ALT, alanine transaminase; AST, aspartate transaminase; BUN, blood urea nitrogen; EGFR, estimated glomerular filtration rate; HDL, high-density lipoprotein; LDL, low-density lipoprotein; VLDL, very-low-density lipoprotein; NA, not applicable. Upward-pointing arrow: higher than normal range. Down-pointing arrow: lower than normal range.

### Reduced fermentation activity in the small intestine.

**FIG 3:**
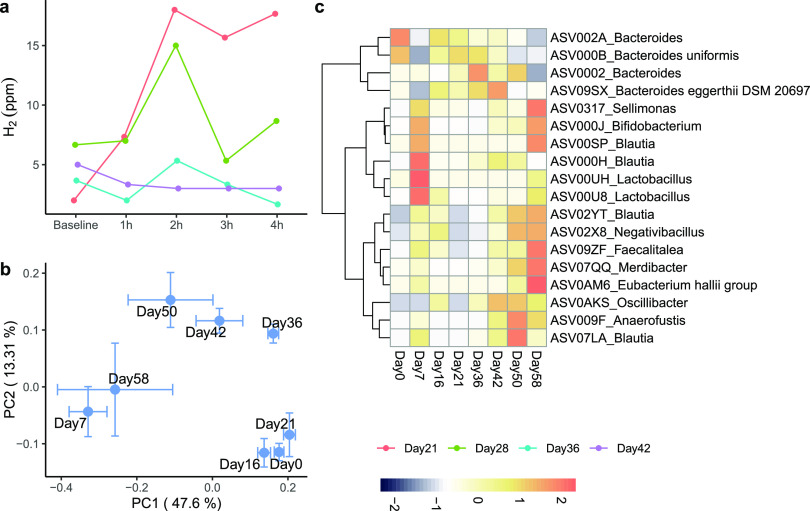
The high-fiber formula changed fermentation in the small intestine and the gut microbial structure. (a) Breath hydrogen levels were measured before and 4 h after taking NBT-NM108 in the morning. (b) Principal-coordinate analysis (PCoA) based on the Bray-Curtis distance was performed on stool samples during the 2-month intervention. Each data point represents the mean Principle coordinates (PC) score, and the error bar represents the standard error from triplicates. (c) Relative abundances of ASVs significantly correlated with PC1 and PC2 scores in panel b. The Pearson correlation was calculated with the *P* value adjusted by the Benjamini-Hochberg procedure (an adjusted *P* value of <0.05 is significant). The heat map shows the row-scaled relative abundance for each ASV. ASVs were clustered using Euclidean distance and the Ward.D2 method.

Small intestinal bacterial overgrowth (SIBO) occurs when bacteria are significantly enriched in the small intestine, which could result in symptoms including nausea related to increased pressure from fermentation gas production (13). Here, we considered SIBO a potential mechanism for the long-lasting nausea and loss of appetite in this case. A noninvasive method for diagnosing SIBO is a breath hydrogen test using a fermentation substrate (13). We collected breath samples after high-fiber formula intake at days 21, 28, 36, and 42 and measured hydrogen levels to examine the response of bacteria in the small intestine to the high-fiber formula. The breath hydrogen level first increased, peaked 2 h after, and then stayed high until 4 h after the intake of the high-fiber formula at day 21, indicating high fermentation activity in the small intestine. On day 28, the breath hydrogen level showed a similar trend but with a slightly lower peak and a decrease after the peak. On both days 36 and 42, the breath hydrogen level stayed low for all 4 h after high-fiber formula consumption, with a slight peak at day 36 and no peak at day 42 (Fig. 3a). These changes during this 2-month intervention indicated a decrease in fermentation gas production in the small intestine, which might be associated with the alleviation of the overall severity of nausea and loss of appetite.

### Changes in the gut microbiota during the nutritional intervention.

To explore the possible role of the gut microbiota in mediating the effects of the high-fiber formula on the alleviation of symptoms, we collected fecal samples before the intervention (day 0) and weekly during the 2-month intervention to profile the gut microbiota composition using 16S rRNA gene V4 amplicon sequencing. A total of 283 amplicon sequence variants (ASVs) were identified. Unlike conventional taxon-based analysis that performs taxonomic assignment on each ASV and collapses the ASVs at a particular taxon level, e.g., genus, as units for analysis, we performed further analysis using ASVs directly as individual variables. This reference-free strategy enabled us to analyze the data at the highest resolution, which is down to single-nucleotide differences, so that we do not lose novel ASVs that cannot be assigned proper taxonomic names. We identified key ASVs that not only responded to the intervention but also correlated with symptoms. In the principal-coordinate analysis (PCoA) plot based on the Bray-Curtis distance calculated from the 283 ASVs, the overall gut microbial structure had a remarkable change from day 0 to day 7 along the first principal coordinate (PC1), which explained 47.6% of the total variation. At day 16 and day 21, the microbial structure became similar to that at day 0. Starting from day 36, it had a continuous change along PC1 and eventually had a microbial structure at day 58 similar to that at day 7 (Fig. 3b). Such a forward-backward-forward pattern might be related to the changes in food intake. The severity of the symptom loss of appetite was evaluated based on the food intake amount for that day, with a high number representing low food intake. At the beginning of the intervention, the participant’s food intake was very low (Fig. 2a). Therefore, the high-fiber formula was the main energy source for the gut microbiota for the first week, which might explain the remarkable change along PC1 at day 7. With the improvement of the symptom loss of appetite at days 16 and 21 (Fig. 2a), the participant started to increase her normal food intake, which might have driven the microbiota change to a structure similar to that at baseline. The continued consumption of the high-fiber formula at 3 doses per day for more than 1 month might have changed the microbiota back to the same space as that on day 7.

To find the gut microbial members that responded to the high-fiber formula intervention, we correlated the abundances of ASVs with both the PC1 and PC2 scores in Fig. 3b. Among the 18 ASVs that showed significant correlations (Fig. 3c), 4 *Bacteroides* ASVs were decreased and 14 ASVs belonging to the genera *Oscillibacter*, *Sellimonas*, *Bifidobacterium*, *Blautia*, *Lactobacillus*, *Faecalitalea*, *Anaerofustis*, *Negativibacillus*, *Merdibacter*, and *Eubacterium* were increased by the intervention. Some species in the *Bacteroides* genus are pathogens due to their virulence, being able to adhere to and impair gut tissues and evade host immunity (25). Species in the genera *Oscillibacter*, *Sellimonas*, *Bifidobacterium*, *Blautia*, *Lactobacillus*, *Faecalitalea*, *Anaerofustis*, and *Eubacterium* were shown to be SCFA-producing bacteria (26–37). The promotion of these SCFA-producing bacteria might increase the SCFAs in the gut to protect the gut barrier and prevent antigens such as endotoxins from entering the circulation (38). Such microbiota changes might regulate the immune system by downregulating proinflammatory cytokines and upregulating anti-inflammatory cytokines (39), therefore helping to alleviate the severity of symptoms in this case.

### Associations between the gut microbiota and symptom alleviation.

**FIG 4:**
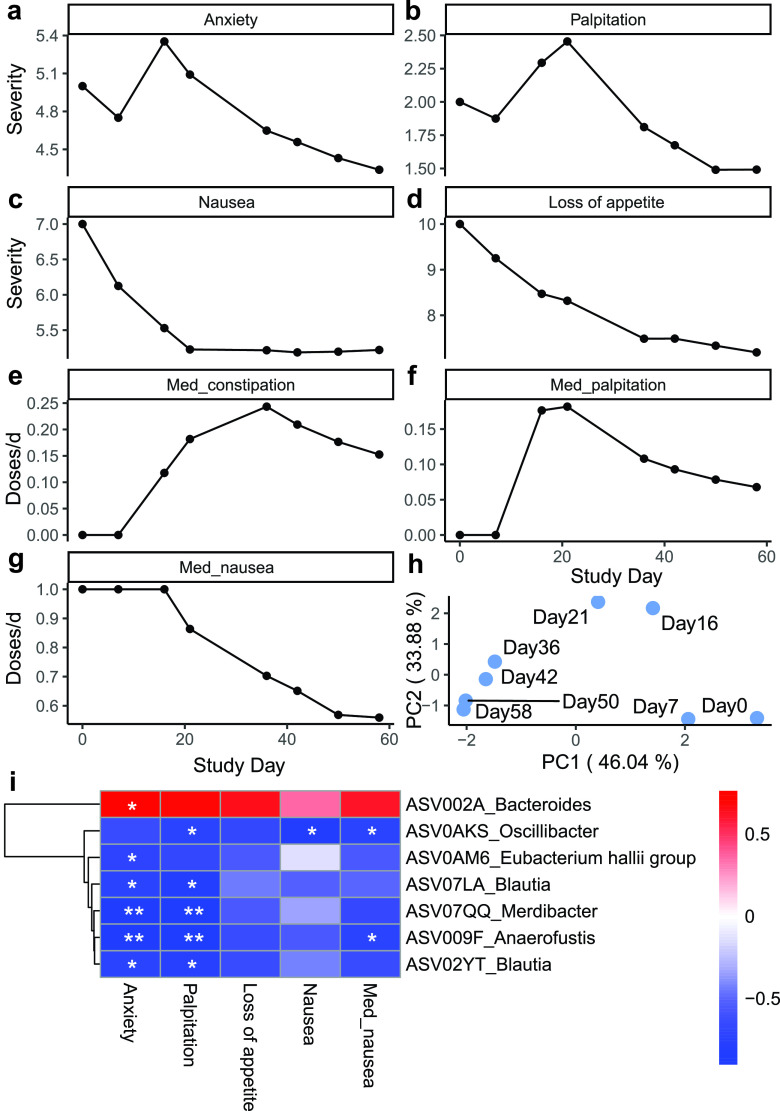
Changed microbial members were associated with the alleviation of symptoms. (a to g) The mean severity of symptoms and the need to take medication decreased by the end of the intervention. The mean severity of symptoms since the start of the intervention was calculated for days 0, 7, 16, 21, 36, 42, 50, and 59, corresponding to the days when the fecal samples were collected. (h) Principal-component analysis (PCA) was performed on the scaled mean severity of symptoms and dosage of medication during the 2-month intervention. Only the time that corresponded to the day of fecal sample collection is shown on the plot. (i) ASVs that were significantly correlated with the mean severity of symptoms. Spearman correlation coefficients are shown as colors in the heat map. Significance is indicated by asterisks (*, *P* < 0.05; **, *P* < 0.01). Med_constipation, dosage of medication for constipation; Med_palpitation, dosage of medication for palpitation; Med_nausea, dosage of medication for nausea.

Since the daily symptoms fluctuated greatly, the average severity of each symptom and the average intake dosage of each medication at days 0, 7, 16, 21, 36, 42, 50, and 58 (days when fecal samples were collected) were calculated by taking the average value from day 0 to the day of interest mentioned above to represent the overall conditions after the intervention. The average severity of anxiety and palpitation increased after day 0, peaked at day 16 and day 21, respectively, and decreased drastically after that (Fig. 4a and b). The average severity of nausea and loss of appetite decreased during the whole intervention period (Fig. 4c and d). The medication intake for palpitation, nausea, and constipation were all instructed to be taken as needed by the physician. The average medication intake for constipation and palpitation slowly increased; peaked at day 36 and day 21, respectively; and decreased after that (Fig. 4e and f). The average medication intake for nausea decreased during the intervention (Fig. 4g). A principal-component analysis (PCA) that combined the average severity of symptoms and average medication intake showed a steady change throughout the intervention from day 0 to day 58 (Fig. 4h). Notably, the gut microbial structure (Fig. 3b) and patient symptoms (Fig. 4h) both showed continuous changes along PC1 from right to left in the PCoA plot and the PCA plot when the intervention was prolonged, which suggests potential associations between the gut microbiota and alleviation of symptoms.

We further correlated the relative abundances of the 18 ASVSs with the average severity of symptoms and medication intake (Fig. 4i). One ASV, ASV002A_*Bacteroides*, was found to be positively correlated with all symptoms and significantly positively correlated with the severity of anxiety. Six ASVs, including ASV0AKS_*Oscillibacter*, ASV009F_*Anaerofustis*, ASV02YT_*Blautia*, ASV07LA_*Blautia*, ASV07QQ_*Merdibacter*, and ASV0AM6_Eubacterium hallii, were negatively correlated with all symptoms and medications and significantly negatively correlated with anxiety and palpitation. The negative correlation between ASVs and anxiety is consistent with previous evidence showing that gut microbiota change/probiotic intake affects anxiety-like behavior in mouse models (40–42). All 6 ASVs described above were found to be beneficial bacteria in previous studies. *Anaerofustis* was shown to be present at a significantly lower abundance in inflammatory bowel disease patients than in age- and gender-matched healthy people (43). A decreased abundance of *Blautia* was shown to be correlated with digestive diseases, including acute fatty liver, constipation, and excessive vomiting during pregnancy (44); Crohn’s disease (45); and mortality in graft-versus-host disease (46). Eubacterium hallii oral intake has shown improved insulin sensitivity and energy expenditure in db/db mice (47), and it is found in a lower abundance in patients with inflammatory bowel disease (48). The *Oscillibacter* abundance has been shown to be higher in healthy subjects than in patients with Crohn’s disease (49). Five of the six ASVs were found to be potential SCFA-producing bacteria. *Anaerofustis* and *Blautia* species are acetic and butyric acid-producing bacteria (26–28), while the Eubacterium hallii group and *Oscillibacter* are butyric acid producers (29, 30). SCFA-producing bacteria have been shown to inhibit potential pathogens and create a healthier microbiota environment (14). All of the above-described results showed that the high-fiber formula modulated the gut microbiota of this patient by increasing beneficial bacteria and decreasing potential pathogens, which was associated with the alleviation of symptom severity.

### Conclusion.

This study shows that high intake of dietary fibers with diverse physicochemical structures significantly alleviated severe GI symptoms in a patient with post-acute COVID-19 syndrome. The significant structural shifts in the patient’s gut microbiota induced by the high-fiber formula, particularly the enrichment of SCFA-producing bacteria, might be associated with the alleviation of both GI and non-GI symptoms such as anxiety and palpitation. Prolonged high-fiber intake might also have reduced bacterial fermentation in the patient’s small intestine. Reduced gut pressure from reduced gas production in the small intestine might work as a potential mechanism for the alleviation of GI symptoms such as nausea and loss of appetite. This study indicates the feasibility of nutritional modulation of the gut microbiota for the alleviation of GI symptoms in patients with post-acute COVID-19 syndrome. However, this single case showed only the feasibility of using high-fiber intervention for alleviating GI symptoms in post-acute COVID-19 syndrome. Randomized controlled trials are needed to establish the clinical efficacy and safety of the high-fiber formula as a potential intervention. More mechanistic studies with host immune and metabolic responses involved are also needed for understanding the relationship between the gut microbiota and post-acute COVID-19 syndrome.

## MATERIALS AND METHODS

### Recruitment and intervention.

The high-fiber formula NBT-NM108 (Notitia Biotechnologies Company, NJ, USA) was given investigational new drug status by the FDA (IND 149631) and is being used in an ongoing phase 2 clinical trial treating acute COVID-19 patients (ClinicalTrials.gov identifier NCT04540406). Both the physician and the patient agreed to the hypothesis that NBT-NM108 may alleviate post-acute COVID-19 syndrome. This single-patient expanded access was approved by the Rutgers Institutional Review Board Office in March 2021, and the patient consented to using this product under IND 155864 given by the FDA. NBT-NM108 was provided as sachets that contain corn bran, wheat bran, sorghum bran, oat bran, inulin, and Fibersol-2 (ADM) (15 g dietary fiber/30-g sachet). The participant was given single-patient expanded access granted by the FDA for NBT-NM108 for 2 months in the study. The participant was instructed to slowly increase the dosage of NBT-NM108 starting from 1 dose/day to 3 doses/day.

### Blood sample collection.

Blood sample-based clinical parameters 4 months prior to the intervention were retrieved from the patient’s medical records from the University Hospital in Newark, NJ. Blood samples were collected on days 9, 30, and 58, and blood sample-based clinical parameters were then measured by LabCorp (NC, USA).

### Breath sample collection.

Breath samples were collected before and hourly after taking the high-fiber formula for 4 h on days 21, 28, 36, and 42. Samples were then mailed back to the laboratory, and the breath hydrogen level was analyzed using BreathTracker Analyzer SC (QuinTron Company, WI, USA) within 3 days of collection.

### Fecal sample collection.

Fecal samples were collected at home using a collection kit (Microsetta Initiative) (DaklaPack, NJ, USA) with minor modifications weekly depending on the participant’s bowel movements. The participant was asked to mail the samples back to the research team on the day of collection. Fecal samples were homogenized by vortexing, aliquoted, and centrifuged for 10 min at 16,000 × *g* upon arrival. The pellets were stored at −80°C until further use.

### DNA extraction.

Fecal sample pellets were processed with a modified Q protocol (50). The modifications include the use of a TissueLyser II instrument (Qiagen, Hilden, Germany) at 25 Hz instead of the FastPrep instrument for the mechanical lysis step and the use of nuclease-free water instead of AE buffer to elute the DNA.

### 16S rRNA gene sequencing.

Hypervariable region V4 of the 16S rRNA gene was amplified using PCR with Ion Torrent barcode-tagged primers (515F and 816R) (51, 52). PCR was performed using a mixture of 10 μL of Platinum SuperFi DNA polymerase (Thermo Fisher Scientific), 1 μL of 10 μM forward primer, 1 μL of 10 μM reverse primer with a unique barcode for each sample, 6 μL of nuclease-free water, and 2 μL of a 10-ng/μL extracted DNA solution. The 20-μL reaction mixture was subjected to the following cycling conditions: 98°C for 30 s for initial denaturation; 98°C for 8 s, 59.6°C for 10 s, and 72°C for 10 s for 30 cycles of denaturation, annealing, and extension; 72°C for 5 min for the final extension step; and a hold at 4°C. The PCR products were then purified using AMPure XP beads (Beckman Coulter, FL, USA) in a 1:1.5 sample-to-bead ratio to remove primer-dimers. All purified amplicons were quantified using a Qubit 4 instrument (Thermo Fisher Scientific), diluted to 30 pM, and pooled into a single library for preparing sequencing chips using the Ion Torrent Chef platform (Thermo Fisher Scientific). The prepared chip was then sequenced using the Ion GeneStudio S5 platform (Thermo Fisher Scientific) according to the manufacturer’s protocol.

### Gut microbiota analysis.

Primer and adapter removal, denoising, and quality filtration of the sequencing data were performed using QIIME 2 to obtain amplicon sequence variants (ASVs) (53, 54). Spurious ASVs were further removed by an abundance-filtering method (55). A phylogenetic tree was built using the commands alignment mafft, alignment mask, phylogeny fastree, and phylogeny midpoint-root to generate the weighted UniFrac metric. A taxonomy assignment was performed using the q2-feature-classifier plug-in based on the silva database (release 132) (56). Sequencing reads of each sample were then rarefied by sampling at 20,000 reads according to the rarefaction curve. Bray-Curtis distances were calculated using QIIME 2. Principal-coordinate analysis (PCoA) based on the Bray-Curtis distance was performed to visualize the intervention effect of the high-fiber formula on the patient’s gut microbiota using the R VEGAN package version 2.5-6 (57). Principal-component analysis (PCA) was performed on the scaled mean severity of symptoms and the dosage of medication during the 2-month intervention using the R stats package 4.1.0 (58).

### Statistical analysis.

ASVs that significantly correlated with PC1 and PC2 scores were selected by Pearson correlation with the Benjamini-Hochberg correction method (adjusted *P* < 0.05) (59). Pearson correlations between the severity of symptoms/dosage of medication and the duration of the intervention/consumption dosage of the high-fiber formula were calculated using the R stats package version 4.1.0, and the general linear regression line and 95% confidence interval were plotted using the R ggplot2 package version 3.3.5 (60). Mean values for the severity of each symptom since the intervention began were calculated for days when fecal samples were collected to represent the average severity after the intervention. Spearman correlations were calculated between the selected ASVs and the mean severity of symptoms/dosage of medication using the R stats package 4.1.0.

### Data availability.

The raw gut microbiota sequencing data have been deposited in the Sequence Read Archive at the NCBI under BioProject accession number PRJNA791007.
